# Classification, biology and entomopathogenic fungi-based management and their mode of action against *Drosophila* species (Diptera: Drosophilidae): a review

**DOI:** 10.3389/fmicb.2024.1443651

**Published:** 2024-10-08

**Authors:** Perumal Vivekanandhan, Kannan Swathy, Pittarate Sarayut, Krutmuang Patcharin

**Affiliations:** ^1^Office of Research Administration, Chiang Mai University, Chiang Mai, Thailand; ^2^Department of Entomology and Plant Pathology, Faculty of Agriculture, Chiang Mai University, Chiang Mai, Thailand

**Keywords:** fruit fly, entomopathogenic fungi, microbial pesticide, biocontrol agents, *Drosophila suzukii*, *Drosophila melanogaster*

## Abstract

This review provides a comprehensive analysis of the classification, biology, and management of *Drosophila* species (Diptera: Drosophilidae) with a focus on entomopathogenic fungi (EPF) as a biocontrol strategy. *Drosophila* species, particularly *Drosophila suzukii*, and *Drosophila melanogaster* have emerged as significant pests in various agricultural systems, causing extensive damage to fruit crops. Understanding their taxonomic classification and biological traits is crucial for developing effective management strategies. This review delves into the life cycle, behavior, and ecological interactions of *Drosophila* species, highlighting the challenges posed by their rapid reproduction and adaptability. The review further explores the potential of EPF as an eco-friendly alternative to chemical pesticides. The mode of action of EPF against *Drosophila* species is examined, including spore adhesion, germination, and penetration of the insect cuticle, leading to host death. Factors influencing the efficacy of EPF, such as environmental conditions, fungal virulence, and host specificity, are discussed in detail. By synthesizing current research, this review aims to provide valuable insights into the application of EPF and to identify future research directions for enhancing the effectiveness of EPF-based control measures against *Drosophila* species.

## 1 Introduction

*Drosophila* species, commonly known as fruit flies, belong to the family Drosophilidae and are widely recognized for their significance in both scientific research and agriculture. There are over 1,500 species within the *Drosophila* genus, with *Drosophila melanogaster* being the most extensively studied model organism in genetics, developmental biology, and neuroscience. These small flies are usually about 2–4 mm in length and are characterized by their rapid life cycle, which can complete in as little as 10 days under optimal conditions (Butt et al., 2016). In the wild, *Drosophila* species typically feed on fermenting fruits and organic matter, playing a key role in the decomposition process. However, certain species, such as *Drosophila suzukii* (spotted wing drosophila), have become significant agricultural pests. Unlike most fruit flies that target overripe or decaying fruit, *D. suzukii* can lay eggs in ripening fruit, leading to substantial crop damage. *Drosophila suzukii* Matsumura, known as the spotted wing drosophila (SWD), is a pest of berries and soft-skinned fruits that has spread from Asia to Europe and North America (Butt et al., 2016). The economic impact of *D. suzukii* damage in California alone has been estimated at $39.8 million, with an additional $8 million spent annually on pesticides in the raspberry industry between 2009 and 2014 (Bowen and McDonald, 1999). *D. suzukii* adults are highly migratory and are believed to move between crop and non-crop hosts (Klick et al., 2016). Their movement increases contact with treated plants, exposing them to lethal doses of insecticide, regardless of spray coverage (Noble et al., 2023).

The zero tolerance for fruit infestation by pests has significantly impacted fresh markets, frozen berries, and fruit export programs (Mazzi et al., 2017; Asplen et al., 2015). Larval feeding inside the fruits results in decay, making them unmarketable and lowering their quality when processed. Severe economic losses have been reported worldwide, particularly affecting high-value fruit crops such as raspberries, blackberries, blueberries, strawberries, and sweet cherries (De Ros et al., 2015). To minimize fruit loss, broad-spectrum insecticides are applied multiple times during the season (Van Timmeren and Isaacs, 2013; Shawer et al., 2018a).

Larval feeding is the primary cause of fruit deterioration. Puncturing the fruit peel also facilitates secondary infections by bacterial and fungal pathogens (Walsh et al., 2012). This pest has been observed infesting a wide range of fruits and vegetables, including blackberries, blueberries, cherries, peaches, raspberries, strawberries, grapes, and kiwis (Kanzawa, 1939; Bolda et al., 2010; Lee et al., 2011). More than 50 wild host plants have been identified in Europe and the United States, providing the pest with a diverse reservoir of alternative hosts throughout the seasons (Baroffio et al., 2015). *Drosophila* species are found globally, thriving in various environments due to their adaptability and reproductive capacity. They are also known for their ability to disperse rapidly, making them a challenge to control in agricultural settings. Understanding the biology and behavior of *Drosophila* species is crucial for developing effective management strategies, especially for those that pose a threat to fruit production.

Current chemical control of *Drosophila* species, particularly *Drosophila suzukii*, relies heavily on the use of insecticides, such as organophosphates, pyrethroids, and spinosyns. These chemicals are applied to protect fruit crops from infestation, often requiring frequent applications due to the rapid life cycle of these pests. While effective in reducing populations, chemical control has several significant disadvantages.

Over time, the frequent use of insecticides can lead to the development of resistance in Drosophila populations, diminishing the long-term efficacy of these treatments. Additionally, the non-selective nature of these chemicals poses risks to non-target organisms, including beneficial insects like pollinators and natural predators. The accumulation of chemical residues on fruits also raises concerns for consumer health and can lead to market rejections, particularly in regions with strict pesticide residue regulations. Furthermore, the environmental impact of repeated pesticide use, including potential contamination of soil and water, highlights the need for more sustainable pest management alternatives.

Entomopathogenic fungi (EPF) offer a promising, eco-friendly alternative to chemical insecticides for controlling *Drosophila* species. These fungi naturally infect and kill pests through spore adhesion and cuticle penetration, reducing reliance on harmful chemicals while minimizing risks to non-target organisms, environmental contamination, and pesticide resistance development. Entomopathogenic fungi (EPF) infect and kill insects by using them as hosts to complete a stage of their life cycle (Vivekanandhan et al., 2018a; Shin et al., 2020; Swathy et al., 2024a,b; Vivekanandhan et al., 2024a, 2024b, 2024c, 2024d). EPF is a successful pest management strategy in agriculture worldwide (Perumal et al., 2024a, 2024b) and is effective against various fruit fly species (Quesada-Moraga et al., 2006; Yousef et al., 2017; Oreste et al., 2015). As biological control agents, EPF can serve as alternatives to chemical pesticides (Tarasco et al., 2016). However, the virulence of entomopathogens varies significantly among species (Oreste et al., 2012). They offer potential for controlling fruit flies in the soil during adult and pupal stages, as well as in fruits during the larval stage (Oreste et al., 2015; Yousef et al., 2017).

Four fungal classes Ascomycota, Basidiomycota, Microsporidia, and Zygomycota contain entomopathogenic species (Bergman et al., 2019). EPF can also act as endophytes, antagonists of plant pathogens, and promoters of plant growth (Vega et al., 2008). The diversity of EPF offers great prospects for integrated pest management (Vivekanandhan et al., 2023a).

Bio-fungicides and bioinsecticides are among the most advanced and widely produced formulations (Swathy et al., 2024a,b). Some of these products, such as those containing entomopathogenic fungi like *Beauveria bassiana* and *Metarhizium anisopliae*, are available commercially (Krutmuang et al., 2023). Due to the restricted use of conventional pesticides and the emergence of new bioproducts, the microbial-based biopesticides market was valued at USD 3.48 billion in 2018 and is projected to reach USD 7.38 billion by 2023 (Singh and Mazumdar, 2022). However, the ecological impact of these products remains unclear.

Several studies have reported positive effects from beneficial interactions with crops (Dara, 2017; González-Pérez et al., 2022), neutral effects on soil and plant microbiome composition (Peng et al., 2021), and negative impacts on certain bees and other insects (Toledo-Hernández et al., 2016). This review aims to analyze *Drosophila* species’ classification, biology, and the efficacy of entomopathogenic fungi as biocontrol agents, focusing on their mode of action and influencing factors.

## 2 Classification

The genus *Drosophila*, within the family Drosophilidae and order Diptera, comprises over 1,500 species, commonly known as *Drosophila melanogaster* and *Drosophila suzukii*. *Drosophila melanogaster* is crucial in genetic and developmental biology research due to its well-documented genome, while *Drosophila suzukii* is known as an agricultural pest (Cini et al., 2012; Perveen, 2018). The genus was first described by Johann Wilhelm Meigen in 1830 and continues to be a focal point in scientific research due to its genetic accessibility and ecological importance.

**Kingdom:** Animalia.

**Phylum:** Arthropoda.

**Class:** Insecta (Insects).

**Order:** Diptera (True Flies).

**Family:** Drosophilidae (Vinegar Flies, Pomace Flies, or Fruit Flies).

**Genus:** *Drosophila.*

### 2.1 Biology of *Drosophila* species

The biology of *Drosophila* species, one of the most extensively studied organisms, is rich and diverse. Its rapid life cycle, progressing from egg to adult in about 10 days, makes it ideal for studying development. The species undergoes complete metamorphosis, with distinct egg, larval, pupal, and adult stages, each offering unique insights into developmental biology. Behaviorally, *Drosophila* species exhibits complex mating rituals, sophisticated feeding habits, and well-defined circadian rhythms, making it a key organism in behavioral research. Genetically, it has been fundamental in understanding inheritance, gene function, and evolution, thanks to its fully sequenced genome. Ecologically, *Drosophila* species are adaptable and inhabit diverse environments, playing crucial roles in ecosystems. Their biology continues to provide critical insights across multiple scientific disciplines (Hoikkala and Poikela, 2022).

### 2.2 Life cycle and development of *Drosophila melanogaster* and *Drosophila suzukii*

**Figure 1:**
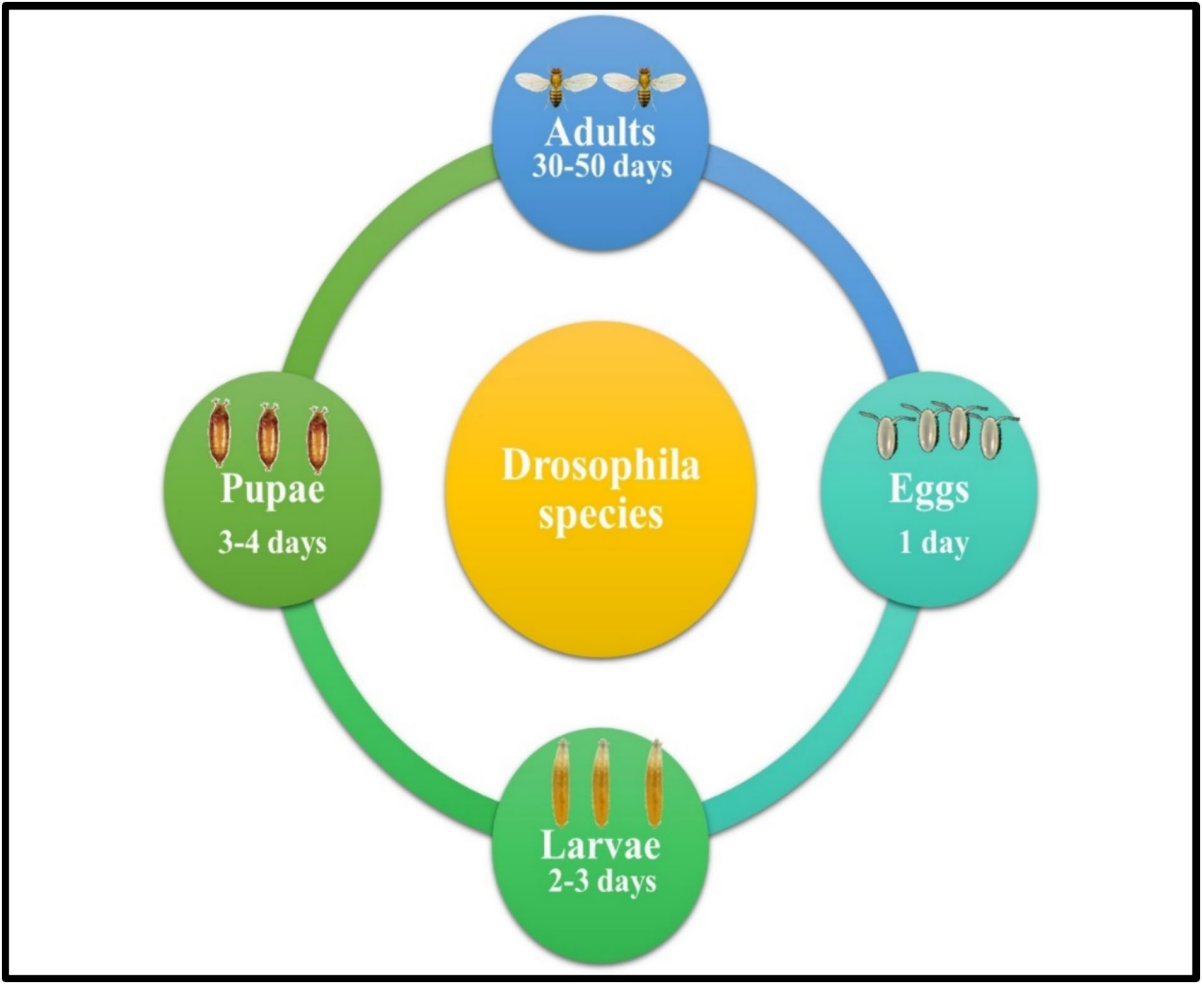
Life cycle of *Drosophila* fruit fly species.

***Egg Stage***: Female *Drosophila* lay their eggs in moist environments abundant with decaying organic material, such as overripe fruit or compost. These conditions provide an ideal habitat for the eggs. Within 24 h of being laid, the eggs hatch, releasing larvae that will immediately begin feeding on the surrounding microorganisms (Figure 1).

***Larval Stage***: The larvae progress through three distinct instar stages over a period of 2–3 days (Figure 1). During each stage, they vigorously consume microorganisms and organic matter within their environment, such as decaying fruit. This feeding behavior supports their rapid growth and development, preparing them for the subsequent pupal stage.

***Pupal Stage***: After the larval stage, the *Drosophila* transitions into the pupal stage, a critical phase where metamorphosis takes place. The pupal case gradually hardens and darkens, providing protection as the organism undergoes dramatic internal changes. Over the next 3–4 days, the adult structures, including wings, legs, and eyes, develop and form (Figure 1).

***Adult Stage***: The adult *Drosophila* emerges from the pupal case, fully developed and ready to begin its brief but active life, typically lasting several weeks. Within 8–12 h of emergence, females are capable of mating and begin laying eggs, rapidly initiating the next generation and ensuring the species’ continued propagation (Figure 1).

*Drosophila melanogaster* offers advantages over vertebrate models, including faster growth, shorter life cycles, easier manipulation, lower maintenance costs, and fewer ethical and regulatory issues (Alarco et al., 2004; Lionakis and Kontoyiannis, 2010). The fruit fly primarily infests fruits, causing damage and spreading. Biological control of *Drosophila* species has traditionally focused on predators and parasites, but microorganisms and plant extracts are also important now (Schmutterer, 1990).

*Drosophila suzukii* thrives at temperatures from 10 to 30°C, with an optimal range of 18 to 25°C. It does not reproduce or shows decreased activity at temperatures below 10°C or above 30°C, where emerging males become infertile (Dalton et al., 2011). Under ideal conditions, it can complete 3 to 15 generations per year (Kanzawa, 1939). Females lay 7 to 16 eggs daily, continuing for 10 to 59 days per generation.

According to Kanzawa (1939), female *D. suzukii* lay 350 to 400 eggs during their lifetime. Eggs hatch in 1 to 3 days, and larvae mature in 5 to 7 days, often pupating inside fruits. The pupal stage lasts 4 to 15 days. At 25°C, the full life cycle from egg to adult is around 7–10 days, while at 15°C, it takes approximately 21–25 days (Kanzawa, 1939). During summer, with peak reproduction, the population is about 90% juvenile stages (eggs, larvae, and pupae) and 10% adults (Wiman et al., 2014; Emiljanowicz et al., 2014; Grassi et al., 2018). *D. suzukii* can survive harsh winters and avoid detection, emerging in non-crop areas in spring and early summer.

*Drosophila suzukii* exhibits seasonal polyphenism, with smaller flies and reduced pigmentation in summer compared to winter (Hoffmann, 2003; Shearer et al., 2016). Larvae do not grow below a certain temperature threshold, but oviposition can occur at temperatures below 10°C (Rendon and Walton, 2019). Debate continues about adult overwintering success in northern climates (Rossi-Stacconi et al., 2016; Bal et al., 2017; Panel et al., 2018).

Current understanding suggests that *D. suzukii* forages during winter when temperatures permit, requiring a food source such as saprotrophic fungi (Stockton et al., 2019a). Dispersal includes both short- and long-distance migrations influenced by phenology, nutrition, and environmental conditions. During the growing season, the spread of *D. suzukii* is influenced by non-crop hosts near cultivated areas (Klick et al., 2016; Leach et al., 2019). Distribution patterns and migration to higher altitudes for better resources in spring and summer are also observed (Tonina et al., 2016; Tait et al., 2018).

### 2.3 Genetics and genomics

*Drosophila melanogaster* has a well-characterized genome consisting of approximately 14,000 genes across four pairs of chromosomes (Piergentili, 2010; Guruharsha et al., 2011). As one of the first organisms to have its genome fully sequenced, it has become a cornerstone of genetic research. Its relatively simple genome, combined with advanced genetic tools, makes it an indispensable model organism for studying gene function, genetic variation, and evolutionary processes. This genomic information has led to significant discoveries in genetics, developmental biology, and disease research.

The genetic makeup of *Drosophila melanogaster* provides a powerful platform for studying gene function, inheritance patterns, and mutations (Arias, 2008). Techniques such as gene knockouts and transgenesis are used to analyze gene roles and functions, while CRISPR/Cas9 enables precise genome editing to explore genetic mechanisms (Trivedi, 2021).

### 2.4 Behavior

***Mating Behavior***: Male *Drosophila* species perform a courtship dance to attract females, characterized by wing vibrations that produce species-specific songs. This behavior includes visual displays, such as wing positioning, and the release of pheromones. Successful mating depends on the interplay of visual, chemical, and auditory cues (Mitoyen et al., 2019).

***Feeding Behavior***: *Drosophila* species primarily feed on yeast and microorganisms in decaying fruit, which provides essential nutrients for their growth and development (Bing et al., 2018). Their sensitive olfactory system, equipped with specialized sensory neurons in their antennae, allows them to efficiently locate these food sources.

***Circadian Rhythms***: *Drosophila* species exhibit circadian rhythms, internal biological cycles that regulate behaviors such as sleep, feeding, and mating over a 24-h period. These rhythms synchronize with environmental light and dark cycles, promoting survival and reproductive success (Johnson et al., 2023).

### 2.5 *Drosophila melanogaster* and *Drosophila Suzukii*: bio-ecology and habitat

*Drosophila* species are highly adaptable and can be found globally, thriving in diverse environments from tropical rainforests to temperate zones (Throckmorton, 1975). They are commonly associated with decaying fruit, which provides a breeding ground and food source, essential for larval development (Throckmorton, 1975).

Some species, like *D. suzukii*, have adapted to exploit fresh fruit, leading to significant ecological and economic impacts. Known as the spotted wing drosophila, it is a major agricultural pest affecting soft-skinned fruits such as berries and cherries. *D. suzukii* lays its eggs in the fruit, where the larvae cause substantial damage, resulting in economic losses for farmers and necessitating management strategies (Throckmorton, 1975). Beyond agriculture, *Drosophila* species are important for studying ecological interactions, serving as prey for various predators and involved in research on resource competition and microbial community interactions. Their adaptability and ecological roles make them valuable models for understanding ecological processes and evolutionary biology (Throckmorton, 1975).

### 2.6 Immune system and pathogenesis

*Drosophila* species have a sophisticated innate immune system that defends against bacteria, fungi, and viruses (Brennan and Anderson, 2004; Govind, 2008). This system includes both cellular and humoral responses. Cellular immunity involves phagocytosis, where immune cells engulf and digest pathogens. Humoral immunity features the production of antimicrobial peptides, which are secreted into the hemolymph to neutralize and kill microorganisms.

*Drosophila* species is also a valuable model for studying host-pathogen interactions. Researchers use it to investigate how entomopathogens that specifically target insects interact with their hosts, evade the immune system, establish infections, and cause disease (Younes et al., 2020). These studies enhance our understanding of insect immunology and contribute to the development of biological control strategies and broader research on immune responses and pathogen interactions (Ortiz-Urquiza et al., 2015).

### 2.7 Reproduction and population dynamics

Female *Drosophila* species are highly fecund, laying hundreds of eggs over their several-week lifespan. Their short generation time around 10 days from egg to adult supports rapid population growth under optimal conditions. This rapid lifecycle allows *Drosophila* species to quickly exploit resources and adapt to environmental changes (Markow, 2015). In laboratory settings, these traits are advantageous for genetic studies, enabling observation of evolutionary processes and genetic variations across multiple generations.

Population dynamics in *Drosophila* species are influenced by environmental factors such as temperature, food availability, and predation (Khaliq et al., 2014; Wiman et al., 2016). Temperature affects development and metabolic rates, while food availability impacts growth and reproduction (Savage et al., 2004; Burton et al., 2011). Predation pressures influence survival rates and population stability. Researchers study these dynamics to explore evolutionary biology principles, including natural selection, genetic drift, and adaptation mechanisms (Abrams and Matsuda, 1997; Cortez and Patel, 2017).

## 3 Current control methods and their disadvantage

Current chemical control methods for managing *Drosophila* species, particularly *D. suzukii* (spotted wing drosophila), rely heavily on the application of broad-spectrum insecticides. These insecticides, including organophosphates, pyrethroids, and spinosyns, are widely used due to their rapid knockdown effect and broad efficacy against a variety of insect pests. However, despite their effectiveness in reducing *D. suzukii* populations, these chemical control methods present several significant disadvantages that challenge their long-term sustainability and effectiveness.

One of the primary disadvantages of chemical control is the development of insecticide resistance. Continuous and repeated use of the same classes of insecticides can lead to the selection of resistant *D. suzukii* populations. Resistance development has been reported in other pest species subjected to similar management practices, and there is a growing concern that *D. suzukii* could follow the same trend (Bruck et al., 2011). The development of resistance not only reduces the effectiveness of chemical treatments but also necessitates higher doses or more frequent applications, further exacerbating the issue and increasing the financial burden on growers.

Another significant disadvantage of chemical control methods is the negative impact on non-target organisms, including beneficial insects such as pollinators and natural enemies of pests. Broad-spectrum insecticides do not discriminate between target pests and beneficial species, leading to a reduction in the populations of predators and parasitoids that naturally help control *D. suzukii* (Desneux et al., 2007). This disruption of ecological balance can result in secondary pest outbreaks, where other pest species, previously kept in check by natural enemies, become problematic due to the diminished populations of these beneficial organisms.

Moreover, the reliance on chemical insecticides poses serious environmental concerns. The widespread use of these chemicals can lead to contamination of soil and water bodies, adversely affecting aquatic life and overall ecosystem health. Pesticide residues can persist in the environment, leading to long-term ecological damage and potential bioaccumulation in the food chain (Roubos et al., 2014). These environmental risks are particularly concerning in regions with intensive agricultural practices where repeated pesticide applications are common.

Human health risks associated with chemical control methods also represent a major disadvantage. Pesticide exposure, either through direct contact during application or through the consumption of pesticide residues on fruits, can pose health risks to farmworkers and consumers. Chronic exposure to certain insecticides has been linked to various health issues, including neurological disorders, respiratory problems, and even cancer (Mostafalou and Abdollahi, 2017). This concern has led to stricter regulations and Maximum Residue Limits (MRLs) for pesticides, complicating compliance for growers and potentially limiting market access for their products.

Finally, the economic implications of chemical control are significant. The cost of purchasing and applying insecticides, combined with the potential need for multiple applications throughout the growing season, can be substantial. In regions where *D. suzukii* is prevalent, growers may need to apply insecticides as frequently as every 7 to 10 days to protect their crops (Van Timmeren and Isaacs, 2013). This not only increases production costs but also places a financial strain on smaller farms that may struggle to afford such intensive pest management programs.

## 4 Alternative control methods with entomopathogens

The use of entomopathogens as alternative control methods for *Drosophila* species, particularly *D. suzukii*, has gained increasing attention as growers seek more sustainable and environmentally friendly pest management strategies. Entomopathogens, including fungi, bacteria, viruses, and nematodes, offer promising potential as biological control agents due to their ability to target specific insect pests while minimizing harm to non-target organisms and the environment.

Entomopathogenic fungi, such as *B. bassiana* and *M. anisopliae*, are among the most widely studied for controlling *Drosophila* species. These fungi infect and kill their hosts by penetrating the insect cuticle and proliferating within the insect’s body, ultimately leading to the insect’s death (Vivekanandhan et al., 2018a, 2020a,b; Perumal et al., 2024a,b). Research has shown that *B. bassiana* and *M. anisopliae* can effectively reduce *D. suzukii* populations in laboratory and field settings. For instance, Kim et al. (2020) demonstrated that these fungi significantly reduced the survival rates of *D. suzukii* adults when applied to infested fruits. Moreover, entomopathogenic fungi can persist in the environment, providing longer-term pest suppression compared to chemical insecticides.

Another promising group of entomopathogens for *Drosophila* species control are entomopathogenic nematodes (EPNs), particularly species from the genera *Steinernema* and *Heterorhabditis* species. These nematodes are capable of infecting and killing *D. suzukii* larvae and pupae by releasing symbiotic bacteria that cause septicemia in the host. EPNs offer the advantage of being applied directly to the soil, where they can target pupating larvae, a life stage often protected from other control measures.

Bacterial entomopathogens also present opportunities for *Drosophila* species control. *Bacillus thuringiensis* (Bt), a well-known entomopathogenic bacterium, produces toxins that are lethal to various insect larvae upon ingestion. Although Bt is primarily used against Lepidoptera and Coleoptera, some studies have explored its potential against *Drosophila* species. While its effectiveness against *D. suzukii* has been limited compared to other pests, there is ongoing research into optimizing Bt formulations and delivery methods for better control of *Drosophila* larvae in fruit crops (Lacey et al., 2015).

Viruses, such as *Drosophila* C virus (DCV) and *Sigma* virus, are naturally occurring pathogens in *Drosophila* species populations and can cause high mortality rates under certain conditions. These viral pathogens are typically spread through contact or ingestion, making them suitable candidates for biological control when harnessed and applied appropriately. However, the application of viral entomopathogens in field settings remains challenging due to factors such as environmental stability and the need for precise delivery mechanisms (Riddiford and Truman, 2013).

Among the entomopathogens the entomopathogenic fungi offers several advantages over traditional chemical control and other entomopathogens based control methods. First, entomopathogenic fungi are often highly specific to their target pests, reducing the risk of harming beneficial insects, pollinators, and other non-target organisms. Second, they can be applied in various forms, including sprays, soil drenches, and bait formulations, allowing for flexible and targeted application strategies. Third, entomopathogenic fungi are generally considered safe for humans and the environment, making them suitable for use in organic farming systems and other environmentally sensitive areas (Vega et al., 2009).

## 5 Entomopathogenic fungi-based insect pest control

Estimates of fungal species on Earth range from 1 to 12 million (Wu et al., 2019). Of the approximately 100,000 known fungi, 750 to 1,000 are entomopathogenic fungi (Blackwell, 2011; Mora et al., 2018; Wu et al., 2019). Fossil evidence of *Paleoophiocordyceps coccophagus* from Myanmar, dating back 100–110 million years, suggests that EPF have been associated with insects since the Cretaceous period (Sung et al., 2008). EPF play a critical role in regulating natural insect populations and are valuable in integrated pest management (Wang et al., 2017; Perumal et al., 2023b; Vivekanandhan et al., 2023b). They have been successfully used to control arthropod pests (Ekesi et al., 2005; Faria and Wraight, 2007; Vivekanandhan et al., 2022a,b,c,d).

Some opportunistic fungal infections found in deceased insects, such as *Alternaria*, *Aspergillus*, *Cladosporium*, and *Penicillium* species, were initially thought to be generalist fungi rather than specialized entomopathogens (Humber, 2005; Logeswaran et al., 2019; Balumahendhiran et al., 2019). These fungi, with small conidia (2 μm), are widely distributed in soil, air, water, and other substrates (Visagie et al., 2014). EPF are facultative parasites with high survival rates (Litwin et al., 2020). They belong to various taxonomic groups: Oomycetes (12 species), Chytridiomycota (65 species), Microsporidia (339 species), Entomophthoromycota (474 species), Basidiomycota (238 species), and Ascomycota (476 species; Woo et al., 2020). This diversity reflects the broad range of biology, phylogeny, morphology, and ecology within the EPF group (Litwin et al., 2020).

### 5.1 Metarhizium species

*Metarhizium* species (Hypocreales: Clavicipitaceae) are a genus of ascomycete fungi known for their role as biocontrol agents against various insect species (Peng et al., 2022; Perumal et al., 2023a,b; Perumal et al., 2024a,b; Swathy et al., 2024a,b). These fungi are commonly found in soils and plants, where they act as soil saprophytes, rhizosphere microorganisms, endophytes, and pathogens of insects and plants (Vivekanandhan et al., 2021; Vivekanandhan et al., 2020a; Vivekanandhan et al., 2020b). Recent taxonomic revisions have included *Metarhizium pingshaense*, *M. anisopliae*, *M. robertsii*, *M. brunneum*, *M. majus*, *M. guizhouense*, *M. lepidiotae*, *M. acridum*, and *M. globosum* (Peng et al., 2022; Raven et al., 2019). The distribution of these species is highly variable. *M. robertsii* and *M. anisopliae* can infect a wide range of hosts, including wireworms, plants (as endophytes), fruit flies, and herbivorous arthropods (Vivekanandhan et al., 2020a; Vivekanandhan et al., 2020b; Peng et al., 2022).

*M. anisopliae* and *M. robertsii* have been shown to infect *D. melanogaster* (Lemaitre et al., 1997; Gottar et al., 2006; Peralta-Manzo et al., 2014; Hunt et al., 2016). Research has focused on their virulence factors, temperature preferences, and pathogenicity in *D. melanogaster* and *D. suzukii*. *M. anisopliae* was the first commercially available entomopathogenic fungus used to control the cereal beetle *Anisoplia austriaca* and the beet weevil *Cleonus punctiventris* (Maniania, 1992). *M. robertsii* has shown various interactions, including use as a biocontrol agent against the root rot fungus *Fusarium solani* f. sp. *Phaseoli*, and as an endophyte promoting plant root growth (Sasan and Bidochka, 2012, 2013; Mukherjee and Vilcinskas, 2018; Sharma et al., 2018). After inoculation with both live and heat-killed *M. robertsii* spores, *D. melanogaster* preferred cooler temperatures, which likely slowed the growth rate of *M. robertsii* (Hunt et al., 2016).

### 5.2 Beauveria species

*Beauveria* comprises several ecologically and commercially significant species, with *B. bassiana* being the most notable (Rehner and Buckley, 2005; Vivekanandhan et al., 2018a, 2020a,b; Perumal et al., 2024a,b). *B. bassiana* has a broad host range and high heat tolerance, making it an effective microbial control agent (Fargues et al., 1997). Over 65 mycoinsecticide and mycoacaricide products derived from *B. bassiana* and *B. brongniartii* have been registered globally for insect pest control through inoculative applications (Faria and Wraight, 2007). The *Beauveria* genus includes approximately 50 species (Rehner and Buckley, 2005) and has been used in various biological studies involving *D. melanogaster*. Under laboratory conditions, the insecticidal activity of *B. bassiana* has been linked to its higher chitinolytic enzyme activity compared to *Trichoderma* species (Vallejos Sirpa et al., 2014).

The *Beauveria* genus (Ascomycota) is found worldwide and includes four species: *B. bassiana*, *B. brongniartii*, *B. amorpha*, and *B. caledonica* (McGuire and Northfield, 2020). Among these, *B. bassiana* has exceptional pathogenic properties and affects over 700 insect species, including arthropods and flies (McGuire and Northfield, 2020). Infected flies showed that the protein GNBP3 is crucial for fungal pathogenesis and exhibits high inhibitory activity (Ekengren and Hultmark, 1999; Gottar et al., 2006). In *D. melanogaster*, the peptide Destruxin A modulates ion transport in renal and gastrointestinal tissues while also suppressing the innate immune response (Pal et al., 2007). The elevated chitinolytic enzyme activity of *B. bassiana* in laboratory settings is associated with its insecticidal virulence.

### 5.3 Isaria species

In 2005, the genus *Isaria* (Ascomycota) was taxonomically distinguished from *Paecilomyces* (Luangsa-ard et al., 2005). The genus currently includes four species: *I. cateniannulata*, *I. fumosorosea*, *I. javanica*, and *I. farinosa*. These species have been extensively studied and utilized as mycoinsecticides, for example, to manage aphids and whiteflies (Luangsa-ard et al., 2005). Members of this group typically have ellipsoidal to fusiform-elliptical conidia (Zimmermann, 2008). However, there is limited information in the literature regarding the metabolites and toxins produced by *Isaria* species (Zimmermann, 2008).

### 5.4 Verticillium species

In 2001, the genus *Lecanicillium* (previously *Verticillium*) was reclassified to include five species: *L. lecanii*, *L. longisporum*, *L. attenuatum*, *L. muscarium*, and *L. nodulosum* (Goettel et al., 2008). These species have been isolated from various insects and target a range of hosts (Goettel et al., 2008).

### 5.5 Entomophthora species

Entomophthora (Entomophthoromycota) comprises 21 highly host-specific species that cause epizootics in insects, including beetles, flies, midges, aphids, fungus gnats, and bugs (Elya and De Fine Licht, 2021). The most well-known species, *E. muscae*, primarily parasitizes adult houseflies (*Musca domestica*). Although Entomophthora is generally host-specific, in laboratory conditions, species like *E. muscae* have been shown to infect a variety of hosts, including *D. melanogaster* (Elya et al., 2018) and *D. suzukii* (Becher et al., 2018).

Flies infected with *E. muscae* laid fewer eggs at lower temperatures, suggesting that the fungus is more effective at lower temperatures (Koger et al., 2020). *E. muscae* also induced cold-seeking behavior in *M. domestica*, resulting in elevated spore levels throughout the infection (Kalsbeek et al., 2001). In *D. suzukii*, the fungus was effective at 23°C, infecting and killing both male and female flies (Becher et al., 2018). This indicates that *E. muscae* might be more effective against insect pests during cooler periods, such as at night and early morning.A recent study found that *D. melanogaster* differed in their genetic susceptibility to *E. muscae*-induced infections (Wang et al., 2020). Similarly, resistance to *M. anisopliae* varied significantly among other wild-type Drosophila verities (Liu et al., 2017).

## 6 Field studies using different FPF against *Drosophila* species

Entomopathogenic fungi (EPFs) have been used to control fruit flies through various methods, including soil inoculation to target pupating larvae (Ekesi and Mohamed, 2011) and cover sprays to target adults (Genre et al., 2009; Daniel and Wyss, 2010). Other methods involve protein baits (Bedini et al., 2018) and attractant-baited autoinoculators (Ekesi et al., 2007). For pests that respond to visual and olfactory stimuli, EPFs can be dispersed using lures to attract fruit flies to a pathogen focus point, where they pick up and spread the inoculum within the population.

Effective pest management using microbial pesticides should consider the ecological context of the biological agent (Evans, 1999). Integrating EPFs into an ecologically focused strategy could enhance long-term fruit fly reduction by facilitating the transport of inoculum from pupation sites in the soil (Ekesi et al., 2002). EPFs can be introduced through inundative or augmentative releases, and foliar sprays are commonly used in conventional management to assess their efficacy against adult fruit flies.

For example, traps in trees treated with *B. bassiana* (Naturalis-L) captured about 23% fewer *Rhagoletis cerasi* flies compared to control trees (Daniel and Wyss, 2010). Flies typically become infected by ingesting pathogen-containing bait or absorbing conidia from treated foliage. Combining EPFs such as *M. anisopliae* and *B. bassiana* (WG-18) with entomopathogenic nematodes (EPNs) like *Heterorhabditis bacteriophora* (*VS* strain) and *Steinernema carpocapsae* (ALL strain) has shown higher mortality rates in *Bactrocerazonata* and *Bactrocera dorsalis* larvae, pupae, and pharate adults in laboratory, glasshouse, and field conditions. The combination of *B. bassiana* and *H. bacteriophora* consistently produced the most effective results (Wakil et al., 2022).

### 6.1 Effectiveness of EPF on *Drosophila Suzukii* and *Drosophila melanogaster*

Several commercially available formulations of entomopathogenic fungi from the genera *Metarhizium*, *Beauveria*, *Lecanicillium*, *Isaria*, and *Paecilomyces* species were tested for their effectiveness against *D. suzukii* (SWD; Cuthbertson et al., 2014; Naranjo-Lázaro et al., 2014; Woltz et al., 2015). When SWD-infested fruit was dipped in field-rate concentrations of *L. muscarium* (Mycotal, 0.1% solution) and *B. bassiana* (Naturalis, 0.3% solution), no significant effect on fly emergence was observed. However, a direct spray of *B. bassiana* caused 44% adult mortality after 7 days (Cuthbertson et al., 2014).

Cossentine et al. (2016) demonstrated that *D. suzukii* adults could easily acquire lethal fungal infections through contact with conidia by walking over contaminated surfaces or interacting with contaminated individuals. Most susceptibility trials use direct sprays to deposit conidia onto potential host organisms, confirming pathogenic infection after exposure to a known concentration of the fungus. However, laboratory contact assays may not accurately reflect how conidia are naturally acquired in the wild.

In direct spray trials, the exact number of conidia that attach to the insect cuticle is unknown since not all applied conidia may adhere. Conidia attachment depends on the inoculum not running off the surface due to excessive carrier use, and the surface must have the required humidity and nutrients for conidia germination (Hajek and St Leger, 1994). Cossentine et al. (2016) confirmed via microscopy that *D. suzukii* acquired conidia after exposure to dried fungi, consistent with other exposure experiments.Of the three fungal isolates tested in temperature trials, *M. brunneum* was the only one that caused significant mortality in flies over a two-week period at 20°C. This isolate’s ability to infect and kill *D. suzukii* at lower temperatures enhances its potential as a field pest management technique.

The higher mortality rates observed at 30°C for all three isolates under high humidity conditions align with previous findings of shorter lifespans of *D. suzukii* adults at this temperature in both field and laboratory settings (Tochen et al., 2014). In our laboratory tests, the most effective isolate, *M. brunneum*, caused significant mortality in *D. suzukii* within 5 days at 30°C. *D. suzukii* is a prolific insect, laying 5–12 eggs per day (Emiljanowicz et al., 2014). Therefore, the ability of a fungal infection to reduce oviposition before the insect die is a critical advantage in pest management.

The fecundity study showed that exposure to *M. brunneum* significantly reduced the *D. suzukii* pupae. From days 5 to 7 after exposure, the average number of pupae produced per female *D. suzukii* was significantly lower than that of the control, indicating that *M. brunneum* infection may affect oviposition. Although the total number of pupae produced by all females in the *M. brunneum* treatment was significantly less than that in the control from days 5 to 14, the number of pupae per surviving female was not significantly different from the control from days 8 to 14. By this time, significant mortality had occurred among the females exposed to *M. brunneum*. The mean number of pupae per female was more influenced by the 10% of surviving, uninfected females, whose fertility was not compromised by the fungal infection (Emiljanowicz et al., 2014).

The study by Cossentine et al. (2016) supports the susceptibility of *D. suzukii* to entomopathogenic fungi (Naranjo-Lázaro et al., 2014; Woltz et al., 2015) and confirms that *D. suzukii* adults can contract lethal infections through contact with dried conidia. The entomopathogenic fungal (EPF) strains tested showed varying levels of activity against different *D. suzukii* host stages, with pupae being more sensitive to EPF treatments than larvae or adults. Additionally, Ramírez-Camejo et al. (2014) found that *D. melanogaster* is susceptible to the virulence of *A. flavus*, an opportunistic pathogen affecting both humans and animals.

## 7 Successful studies showing pathogenicity against *Drosophila* species

Mortality and oviposition are commonly used laboratory criteria to confirm the effectiveness of EPF-biopesticides. The efficacy of these biopesticides against *D. suzukii* (SWD) depends on the EPF species, the targeted life stage of the insect (larvae, pupae, or adults), and factors such as dosage, frequency, and application method. Naranjo-Lázaro et al. (2014) found that adult *D. suzukii* showed varying susceptibility to different *Isaria* and *Metarhizium* strains: *I. fumosorosea* Pf21 had 85% mortality, Pf17 had 60%, and Pf15 had 57.5%, while *M. anisopliae* had only 12.2% mortality.

Cahenzli et al. (2018) demonstrated that commercial EPF-biopesticides significantly impact adult flies, with effectiveness depending on the EPF species/strain and the gender of the host (females are less affected than males). Cossentine et al. (2016) also noted that EPF effectiveness is influenced by dosage and abiotic factors like temperature. Higher dosages (10^8 conidia) resulted in the highest mortality rates for *B. bassiana*, *M. brunneum*, *I. fumosorosea*, and *L. lecanii*, with mortality increasing at higher temperatures (20°C, 25°C, 30°C). Additionally, *M. brunneum* was found to reduce fly fecundity by decreasing oviposition.

Shawer et al. (2018b) showed that *B. bassiana* was highly effective against SWD in both laboratory and field conditions, achieving over 90% efficacy when used with various insecticides in cherry orchards. Although EPF was less effective than chemical pesticides, it still had a significant impact on SWD control. The combined use of EPF with other biological or chemical agents (e.g., nematodes, bacteria) has shown potential compatibility and efficacy (Sharma et al., 2018; Sain et al., 2019). While some biological agents like *T. longibrachiatum* have shown potential in replacing chemical insecticides (e.g., *Leucinodes orbonalis* in India), research on EPFs specifically interacting with *D. melanogaster* is limited. For example, *T. inhamatum* has been studied for its insecticidal and chitinolytic activities (Vallejos Sirpa et al., 2014).

### 7.1 Comparative efficacy and cost of entomopathogenic fungi vs. chemical insecticides in pest management

The application of entomopathogenic fungi (EPF) in real-world agricultural settings offers a sustainable alternative to chemical insecticides (Vivekanandhan et al., 2024a). However, comparative studies of EPF and chemical insecticides in terms of seasonal efficacy and cost-efficiency have revealed distinct differences in performance. Chemical insecticides, known for their rapid action, often provide immediate pest control, but this comes at the cost of environmental degradation, resistance development, and non-target effects. In contrast, EPF, although slower acting, provide long-term pest control with fewer ecological side effects (Shah and Pell, 2003).

In terms of seasonal efficacy, chemical insecticides are highly effective in the short term, often reducing pest populations within hours to days. However, repeated applications are necessary due to the pests’ ability to develop resistance. Over time, this leads to diminishing returns, requiring higher doses or new chemical formulations. On the other hand, EPF show a slower onset of action, typically taking several days to weeks to achieve significant mortality in pest populations (Vega et al., 2009). This delay can be a disadvantage during peak pest infestation periods when immediate control is crucial. However, EPF have been shown to provide residual control, with the ability to persist in the environment and continue infecting pest populations over extended periods, especially when environmental conditions are favorable (Shah and Pell, 2003). This seasonal persistence can reduce the frequency of applications compared to chemical insecticides.

In terms of cost-efficiency, chemical insecticides are initially more affordable due to their widespread availability and established production processes. However, the hidden costs associated with their use, such as the environmental damage they cause, the loss of biodiversity, and the development of pest resistance, make them less cost-effective in the long term (Faria and Wraight, 2007). Moreover, the need for frequent reapplication due to short residual effects increases the total costs over a growing season. EPF, while often more expensive to produce and formulate, may offer greater cost-efficiency in the long run. For example, studies have demonstrated that the use of EPF can lead to sustained pest population suppression with fewer applications, which can translate into lower overall costs in multi-season agricultural operations (Zimmermann, 2007). Furthermore, EPF reduce the need for additional pest management inputs by promoting a balanced ecosystem, leading to enhanced natural enemy populations that contribute to pest suppression.

Comparative studies in agricultural systems have shown mixed results depending on the specific pest and environmental conditions. For instance, in a study comparing EPF and chemical insecticides for the control of aphids, the chemical insecticides provided faster knockdown, but the EPF-treated fields showed longer-lasting pest suppression with minimal non-target effects (Roy and Pell, 2000). Another study on corn borer control highlighted that while chemical insecticides were more effective initially, EPF provided comparable long-term control with fewer environmental risks (Faria and Wraight, 2007; Table 1).

**Table 1:** Mortality of *Drosophila melanogaster* and *Drosophila suzukii* using entomopathogenic fungal isolates and commercially available entomopathogenic fungi-based formulations in direct and indirect methods.

Fungus	Infection through direct or indirect method	Test concentration (spores/mL)	Mortality percentage	Exposure time (in days)	Target stage	Reference
Mortality of *D. melanogaster* and *D. suzukii* using entomopathogenic fungi based commercially available formulations						
*I. fumosorosea*	Direct	1 × 10^6^–1 × 10^9^	57–90	14	adult (*D. suzukii*)	Cossentine et al., 2016
*L. lecanii*	Direct	1 × 10^6^–1 × 10^9^	62–77	14	adult (*D. suzukii*)	Cossentine et al., 2016
*M. brunneum*	Direct	1 × 10^6^–1 × 10^9^	71–100	14	adult (*D. suzukii*)	Cossentine et al., 2016
*B. bassiana*	Direct	1 × 10^6^–1 × 10^9^	54–96	14	adult (*D. suzukii*)	Cossentine et al., 2016
*E. muscae*	Indirect	2.25 × 10^6^	≤27.3	4–8	adult (*D. suzukii*)	Becher et al., 2018
*T. suzukii*	Indirect	10^1^–10^5^ spores/μl	71.2	19	larvae, pupae and adults (*D. suzukii*)	Biganski et al., 2021
*M. anisopliae* and *I. fumosorosea*	Indirect	1 × 10^9^	≥40	7	adult (*D. suzukii*)	Cuthbertson and Audsley, 2016
*E. muscae*	Indirect	2.25 × 10^6^–3.46 × 10^5^	62.9	10	adult (*D. suzukii*)	Becher et al., 2018
*B. bassiana*	Direct sprays	1 × 10^7^	60	14	adult (*D. suzukii*)	Woltz et al., 2015
*P. fumosoroseus*	Direct sprays	1 × 10^7^	31.3	14	adult (*D. suzukii*)	Woltz et al., 2015
*M. anisopliae*	–	1× 10^7^	12.5	–	adult (*D. suzukii*)	Naranjo-Lázaro et al., 2014
*M. anisopliae*	Direct sprays	1 × 10^7^	61.5	14	adult (*D. suzukii*)	Woltz et al., 2015
*I.fumosorosea*	Direct sprays	1×10 ^7^	85, 57.5, 60	–	adults (*D. suzukii*)	Naranjo-Lázaro et al., 2014
Mortality of *D. melanogaster* and *D. suzukii* using Entomopathogenic fungi based commercially available formulations						
*L. muscarium*	Indirect	0.1% solution	93.3–95.3	7	adult (*D. suzukii*)	Cuthbertson et al. (2014)
*B.bassiana*	Direct spray	0.3% solution	44	7	adult (*D. suzukii*)	Cuthbertson et al. (2014)
*B.bassiana*	fruit-dip	–	70–78	7	adult (*D. suzukii*)	Rhodes et al., 2018
*I.fumosorosea*	fruit-dip	–	62–68	7	adult (*D. suzukii*)	Rhodes et al., 2018
*B. bassiana*	Mycotrol-O		80	10	adult (*D. melanogaster*)	Shahrestani et al., 2018
*M. anisopliae*, and *B. bassiana*	Direct and indirect	2 × 10^5^	20	10	larvae (*D. suzukii*)	Ibouh et al., 2019
			60		pupae (*D. suzukii*)	
			38–68		adult (*D. suzukii*)	

EPF strains exhibited varying effectiveness against different life stages of *D. suzukii*, with pupae being more sensitive to EPF treatments than larvae or adults. Variable mortality rates in *D. suzukii* adults treated with EPF have been reported, but these differences are not consistently linked to inoculation methods such as direct spray or fruit dipping (Naranjo-Lázaro et al., 2014). In some studies, *B. bassiana* inhibited both oviposition on uninfected berries and adult emergence from infested berries, while in others, it had no effect (Gargani et al., 2013; Cuthbertson et al., 2014).

The success of *D. suzukii* as an invasive species is partly due to its ability to tolerate a wide range of environmental conditions. It can travel long distances across continents in various life stages within fruit or shipping containers (Rossi-Stacconi et al., 2016). This species has a broad host range, high fertility, and significant potential for both passive and active dispersal (Emiljanowicz et al., 2014; Stockton et al., 2019b; Thistlewood et al., 2019). Interestingly, female *D. melanogaster* is more susceptible to several strains of *B. bassiana* than males. This sexually dimorphic susceptibility is not solely due to external defense and persists even after the flies are injected with the pathogen (Shahrestani et al., 2018).

## 8 Mechanisms of action of entomopathogenic fungi

Fungi penetrate the insect cuticle through non-sclerotized regions, wounds, the trachea, and mouthparts. This process involves mechanical and enzymatic strategies, with the secretion of chitinases, proteases, and lipases facilitating entry into the host. These enzymes, such as those identified in *Beauveria bassiana* (Zimmermann, 2007), degrade the insect cuticle, allowing the fungus to infiltrate the host’s body. *Metarhizium acridum*, another well-known entomopathogenic fungus (EPF), employs similar methods, though the specific enzymes used remain less understood. Once inside the insect, EPF proliferate within the hemolymph, producing asexual spores called blastoconidia, which invade host tissues, causing physiological and pathological damage that typically leads to death within 3–7 days (Zimmermann, 2007; Shahid et al., 2012).

To resist fungal infection, insects activate defense mechanisms such as the release of phenoloxidase, which catalyzes melanization, and the mobilization of hemocytes for phagocytosis and encapsulation. Additionally, insects produce antimicrobial peptides (AMPs) and other defense molecules (Amparyup et al., 2013; Lavine and Strand, 2002). However, fungi have evolved countermeasures. For example, *B. bassiana* and *Metarhizium anisopliae* produce secondary metabolites, including destruxins, that suppress insect immune responses by inhibiting AMP production and interfering with hemocyte activity (Pedrini et al., 2007).

**Figure 2:**
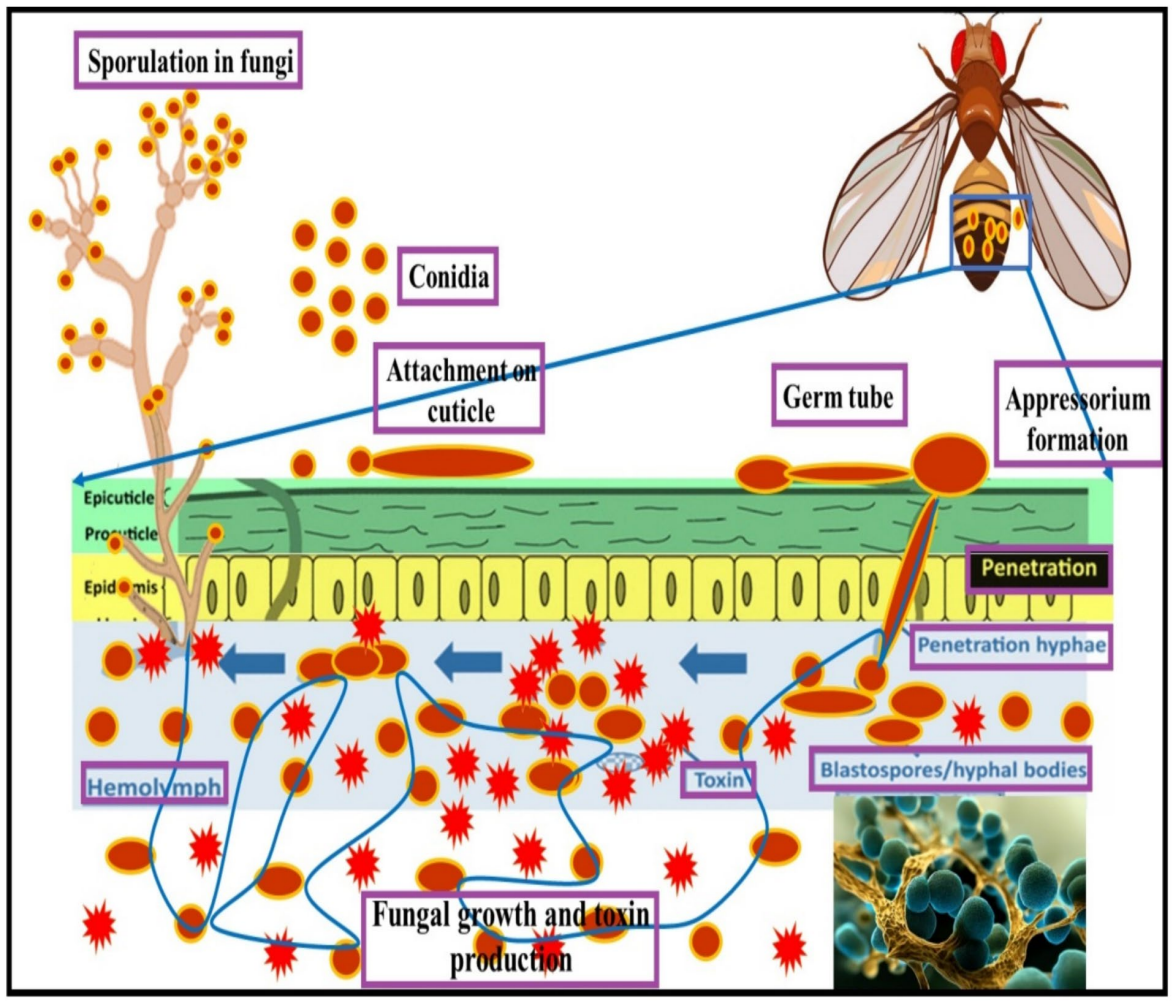
Entomopathogenic fungi mode of infections on *Drosophila* species.

In *Drosophila*, the immune system primarily responds to fungal infections through the Toll pathway, which triggers the production of AMPs like Drosomycin. The Imd pathway, while primarily responsive to Gram-negative bacteria, also plays a role in fungal defense (Lemaitre and Hoffmann, 2007). By inhibiting Toll signaling, fungi like *B. bassiana* evade these immune responses, enhancing infection success (Gillespie et al., 1997). Understanding the molecular mechanisms of these interactions is crucial for optimizing EPF use in pest control strategies (Figure 2).

## 9 Pathogenic prowess: the future of *Drosophila* pest management

In recent years, 58 EPF-biopesticides have been developed and commercialized in various countries, based on the following species: *M. anisopliae* (33 products), *B. bassiana* (20 products), *B. brongniartii* (5 products), *L. lecanii* (2 products), *I. farinosa* (1 product), and *I. fumosorosea* (1 product). Several commercial strains of entomopathogenic fungi (EPF) have been registered with the US Environmental Protection Agency as potential active ingredients, including six strains of *B. bassiana* (Mohammed et al., 2022). The formulation of EPF products significantly impacts their efficacy against insect pests. For example, formulations of *B. bassiana* with plant oils (e.g., Naturalis®) have shown higher mortality rates than those using wettable powders (e.g., Bb-Protec; Cahenzli et al., 2018). Oil formulations, compared to aqueous solutions, can extend the shelf life of conidia and protect them from abiotic stressors such as humidity and temperature (Paixão et al., 2017). These findings highlight the importance of using oil co-formulations in commercial EPF products.

Fungi emit volatile organic compounds (VOCs) as byproducts of metabolism, which can impact other species and human health, though their mechanisms of action are not fully understood (Bennett and Inamdar, 2015; Vivekanandhan et al., 2018b; Vivekanandhan et al., 2018a). Inamdar et al. (2014) used *Drosophila* species as a model organism to study the biological effects of fungal VOCs. They found that eight-carbon VOCs from fungi were significantly more lethal to *Drosophila* species than common industrial solvents like xylene (Inamdar and Bennett, 2014). Flies with immunological deficiencies, caused by mutations in both the Toll and Imd pathways, exhibited greater resistance to eight-carbon VOCs compared to wild-type flies or single mutants, suggesting that these compounds can affect immune responses (Almaliki et al., 2021). This highlights *Drosophila* species as a valuable model system for investigating the effects of volatile fungal metabolites on animals.

Examples of opportunistic entomopathogenic fungi include *Aspergillus*, *Penicillium*, and *Trichoderma* species. Bojke et al. (2018) identified some VOCs from *M. anisopliae* and *B. bassiana*, but their mechanisms of action remain unknown and have not been tested in *Drosophila*. Given the potential for these VOCs to repel and control insect pests or pathogens (Lozano-Soria et al., 2020).

## 10 Eco-friendly solutions: sustainable drosophila control with entomopathogenic fungi

Growers rely on regular insecticide applications to manage adult *D. suzukii* populations in the field. Although new pesticides for controlling this pest in berry crops have been developed, growers often reach the seasonal maximum limit for important insecticides (Chouinard et al., 1992). There is an urgent need for sustainable pest management solutions that complement biological control efforts and reduce reliance on chemical inputs. Alternative methods such as alternating row sprays, border sprays, and mass trapping offer options to the widespread application of pesticides. These approaches aim to minimize damage caused by *D. suzukii* in berry crops, reduce pesticide residues, decrease the volume of pesticide used, and mitigate fruit damage from application equipment (Prokopy et al., 2003; Klick et al., 2016). Border sprays, applied to the field perimeter, help prevent pests from neighboring areas from entering (Blaauw and Isaacs, 2015). Since *D. suzukii* can utilize wild hosts in adjacent woodland areas (Briem et al., 2016; Klick et al., 2016; Iglesias and Liburd, 2017), border sprays could be an effective management strategy. Additionally, Sain et al. (2021) found that strains of *B. bassiana*, *M. anisopliae*, *F. moniliforme*, and *I. javanica* are compatible with several conventional pesticides, including spiromesifen, diafenthiuron, buprofezin, pyriproxyfen, and flonicamid. This compatibility opens new opportunities for integrating EPF into pest management programs in pesticide-exposed areas. Natural substances that act as repellents, toxicants, or deterrents have been primarily tested on *D. suzukii* adults (Dam et al., 2019).

**Figure 3:**
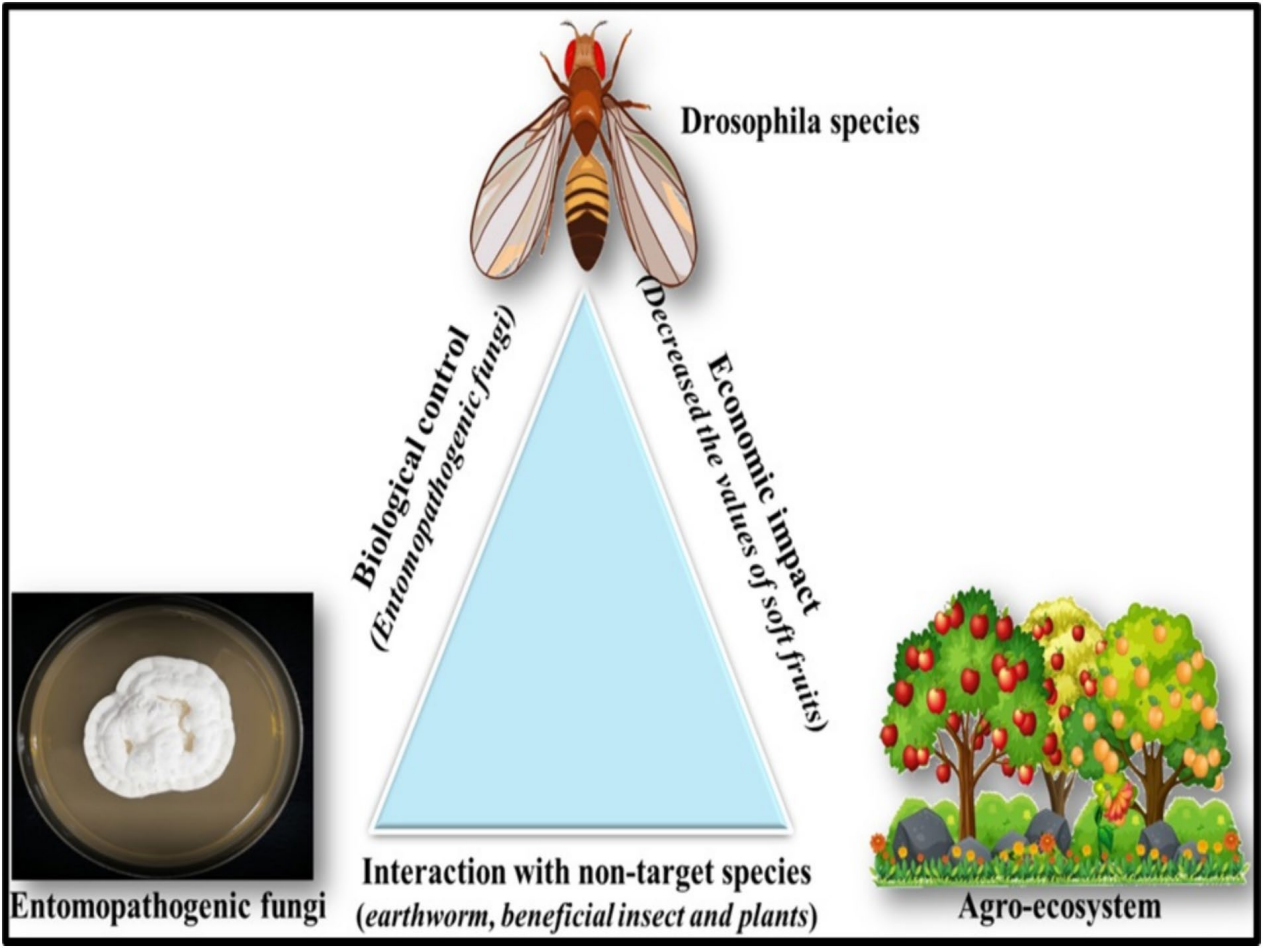
The ecofriendly approaches of using biological control agents like entomopathogenic fungi subsequently enriches the environment and kills the target pest.

Commercial entomopathogenic fungi have shown variable results against *Drosophila suzukii*, potentially due to suboptimal field conditions (Lee et al., 2019). This control strategy could be improved by using indigenous strains better adapted to local environments (Haye et al., 2016; Cossentine and Ayyanath, 2017). For example, BotaniGard, a bioinsecticide containing a high concentration of *B. bassiana*, was tested against *D. suzukii*, a major global pest of soft fruits (Cossentine et al., 2016). Another bioinsecticide, Mycotrol-O, also based on *B. bassiana*, achieved 80% adult mortality in strawberry experimental cages 10 days after application (Jentsch et al., 2014). Alternative EPF-based control strategies include lure-and-infect or lure-and-kill devices (Cossentine et al., 2016; Yousef et al., 2018), which feature fungal spores in baited auto-inoculators that protect the spores from environmental degradation. Trials with a noncommercial fungus strain resulted in 96% mortality after 24 h of exposure, demonstrating its potential for selective and cost-effective management of *D. suzukii* (Yousef et al., 2018). The use of EPF-based agricultural products can introduce EPF into agroecosystems, where they may interact with various organisms, plants, and microorganisms (Figure 3).

The full impact of EPF-based products on ecosystem functioning is not yet fully understood. Key challenges in researching the non-target impacts of EPF in agricultural environments include their persistence and spread in treated fields, the wide range of potential hosts, and interactions with or infections of non-target organisms. EPF persistence in agroecosystems varies due to environmental conditions such as UV radiation, temperature, humidity, and soil factors like pH, texture, organic matter, and rhizosphere composition. Some studies have found that commercial EPF has a shorter persistence in environments like soil, often less than 2 to 3 months (Lei et al., 2022; Yang et al., 2019), which may enhance their potential for field application in pest control.

Researchers are studying the effects of various EPF species on plants, invertebrates, and non-target insects, exploring their synergistic, antagonistic, or neutral interactions. EPF can serve as biopesticides for insects and also as potential biofertilizers or biostimulants, promoting plant growth and enhancing soil nutrient availability (Bamisile et al., 2021). For example, *M. anisopliae* has been suggested as a biostimulant for Arabidopsis, tomato, and maize plants, increasing root length and fresh weight (González-Pérez et al., 2022). *M. robertsii* enhances root growth in Arabidopsis by increasing indole-3-acetic acid production, which also boosts its virulence against *G. mellonella* (González-Pérez et al., 2022). Additionally, *B. bassiana* acts as a biostimulant for cabbages, positively impacting development under water stress conditions (Dara, 2017). *M. brunneum* has been shown to enhance iron availability in calcareous soil and in cucumbers and melons (Barelli et al., 2016; García-Espinoza et al., 2023). EPF strains have generally shown neutral effects on non-target microbiomes and biota.

## 11 Precision biocontrol: strategies for optimized application

Biological control primarily involves using predators or pathogens to regulate the population of Spotted Wing Drosophila (SWD). While promising, the success of using predators, such as sterile insects, has been limited. A well-researched form of biological control is the use of entomopathogenic fungi (EPF), which have proven effective against a range of insects, including *D. suzukii* (Woltz et al., 2015; Cossentine et al., 2016). Consequently, many EPF species are now used as active ingredients in biopesticide formulations. The minimal environmental risk associated with EPF-based biopesticides has generated significant interest in their development and application in agriculture.

### 11.1 Entomopathogenic fungi limitations and challenges in field application

Entomopathogenic fungi (EPF) are promising biological control agents for managing insect pests in sustainable agriculture. However, their field application faces several limitations and challenges that hinder their widespread adoption. One significant challenge is their dependency on environmental conditions. EPF require specific humidity and temperature ranges for successful spore germination and infection. In field conditions, low humidity and extreme temperatures can significantly reduce their effectiveness, as these fungi rely on high moisture levels to penetrate the insect cuticle (Shah and Pell, 2003).

Another limitation is the slow action of EPF compared to chemical insecticides. While chemical pesticides can kill pests within hours, EPF generally take several days to weeks to kill their hosts, allowing pests to continue damaging crops during this period. This delay in action often leads to hesitation among farmers seeking quick solutions (Vega et al., 2009). Additionally, the sporulation and production of infective units in large quantities remain a technical challenge. Ensuring spore viability during storage and application can be problematic, as spores may degrade or lose virulence before reaching the target pests (Zimmermann, 2007).

Formulation and delivery are also complex. Spores need to be formulated in a way that enhances their stability and adherence to the insect cuticle in outdoor conditions, which often proves difficult. Moreover, scaling up the production of high-quality spores for commercial use is expensive and technically demanding, adding to the cost of using EPF compared to conventional insecticides (Faria and Wraight, 2007). Non-target effects also raise concerns, as certain strains of EPF can inadvertently affect beneficial insects, such as pollinators or natural enemies of pests, creating a risk of disrupting ecological balances (Roy and Pell, 2000).

### 11.2 Integration of insect pathogenic fungi into IPM programs

Entomopathogenic fungi (EPF) have been developed as an alternative pest control method in response to concerns about the harmful effects of synthetic pesticides on humans and the environment. As a result, they are increasingly integrated into pest management (IPM) systems. Fungicides, however, can negatively impact EPF by killing these beneficial fungi and contributing to pest outbreaks and resurgence. For instance, fungicides used to treat pecan scab have been shown to kill EPF that control pecan aphids, necessitating additional insecticide applications to prevent secondary outbreaks (Dutcher, 2007; Pickering et al., 1990).

Globally, microbial biopesticides represent approximately US$3.3 billion, about 8% of all pesticides sold (Glare et al., 2020). Their usage is expected to increase in the coming decades (Olson, 2015). EPF is the second most widely used microbial biopesticide, accounting for about 9% of all microbial biopesticides sold worldwide (Glare et al., 2020). Their popularity is due to their effectiveness against a range of insect pests (Dolinski and Lacey, 2007; Lacey and Shapiro-Ilan, 2008) and their suitability for organic and sustainable agriculture (Lacey et al., 2015).

EPF have also been shown to act as endophytes in host plants (McKinnon et al., 2017). They can be combined with attractants to create attract-and-kill pest management strategies (Navarro-Llopis et al., 2015; Brandl and Andersen, 2017). Additionally, EPF may interact synergistically with beneficial arthropods, such as predators, parasitoids, and pollinators (Rossoni et al., 2014; Al Mazra'awi et al., 2006), as well as with other entomopathogens, like bacteria and nematodes (Wraight et al., 2017), and synthetic insecticides (Wraight et al., 2016). Combinations of EPF with attractants, such as methanol/ethanol mixtures, aggregation pheromones, or sex pheromones, have been tested against pests like *Ceratitis sordidus* (Tinzaara et al., 2007), *Ceratitis formicarius* (Lopes et al., 2014), and *Hypothenemus hampei* (Mota et al., 2017). A “sex pheromone” is a female-produced attractant that draws both sexes of the same species to a calling site, increasing the likelihood of mating (Landolt and Phillips, 1997). An “aggregation pheromone” is a male-produced attractant that lures both male and female individuals of the same species to a specific location to enhance mating chances.

The technique come to a thought to researcher Knipling (1955), that is the Sterile Insect Technique (SIT), which was used to control agricultural pest population. It basically relies on growing of individual pest and reproductively sterile them and release them in geographical environment. Procedure for sterile insect techniques here follows:

- Exposing the insects to ionizing radiation, which ultimately causes the germ cell atrophy (Mutation in the sperm and complete ovary atrophy),
- The ionized insects flooded in the agricultural environment,
- when the sterile males meet the wild female the insemination process takes place,
- and the zygote leads to die during embryogenesis and thereby, reduces the population of insects.

Some researchers have concluded that combining Sterile Insect Technique (SIT) with biological control agents can effectively control insect pests (Enkerlin, 2005; Toledo et al., 2017). When *B. bassiana* was combined with a collection pheromone (Cosmolure®—sordidin or (1S,3R,5R,7S)-1-ethyl-3,5,7-trimethyl-2,8-dioxabicyclo [3.2.1] octane), moderate to high mortality of *Ceratitis sordidus* adults was observed in the laboratory (Lopes et al., 2014). However, field trials showed only low to moderate mortality (Tinzaara et al., 2007; Tinzaara et al., 2015).

Further research into biological management strategies is essential for developing an integrated pest management (IPM) program for spotted wing drosophila (SWD). Recent efforts have focused on addressing major invasive pests in North America and Europe (Ragsdale et al., 2011; Zappala et al., 2013). While biological control of SWD may be effective in reducing populations in natural reservoir habitats, it may not have the same impact on cultivated crops, despite the fly’s high reproductive potential and multiple generations per year.

## 12 Next,-generation tools: developing entomopathogenic fungi and future directions and research gaps

During the internal growth stage, the insect intestine is compromised (Toledo et al., 2010), leading to alterations in the gut microbial community and significant changes in the composition and concentration of host hemolymph metabolites involved in the immune response (Hernández-Chávez et al., 2017; Shin et al., 2020). During host-entomopathogenic fungus (EPF) interactions, many fungal secondary metabolites (SMs) are produced from primary metabolite pools and major metabolic pathways. These secondary metabolites vary dynamically depending on the type of fungal infection and play an important role in fungal development and interactions with other species (Woo et al., 2020; Zhang et al., 2020; Pedrini, 2022). Many of these fungal secondary metabolites, also known as mycotoxins, are toxic to insects. For example, beauverolides, cytochalasins, destruxins, and oosporeins are essential for complete fungal virulence. They suppress or evade the host immune response and can cause muscular damage, such as destruxin A’s action on *Locusta migratoria* visceral muscles, allowing fungal multiplication in insect hosts (Vilcinskas et al., 1997; Feng et al., 2015). These mycotoxins are often specific to certain species, hosts, or infection stages and show promise for biological pest control (Mannino et al., 2021).

Several metabolites derived from entomopathogenic fungi (EPF) influence insect innate immunity. For instance, cordycepin (3′-deoxyadenosine), a secondary metabolite produced by the hypocrealean entomopathogenic fungus *Cordyceps militaris*, reduces immune-related gene expression in insects (Woolley et al., 2020). *Metarhizium acridum* produces tryptamine, which induces the generation of reactive oxygen species in the host while suppressing the immune system by activating a host aryl hydrocarbon receptor (LmAhR; Tong et al., 2020). Further research is needed to explore the roles of fungal metabolites in regulating insect host immunological capacity and gut microbiome features. Specifically, the molecular biology of insect-fungal interactions and the mechanisms by which insect antagonists limit secondary metabolite production in model fungal diseases like *Metarhizium* species remain unresolved (Xu, 2016; Wang and Wang, 2017).

Future research should focus on insect-EPF interactions using molecular and biochemical methodologies, such as metagenomic and metabolomic techniques. Understanding pathogenicity mechanisms will aid in the development of novel biological formulations. Future efforts should prioritize: (a) reducing the lethal dose and application time, (b) enhancing EPF formulation tolerance to various climatic conditions (e.g., low/high humidity, temperature, and UV), and (c) improving EPF tolerance to agrochemicals (e.g., pesticides/insecticides). Enhancing EPF formulations will increase their effectiveness in agricultural systems and integrated pest management (IPM) methods.

## 13 Conclusion

The classification and biological understanding of entomopathogenic fungi (EPF) highlight their significant potential in managing *Drosophila* species. This review underscores the diverse modes of action of EPF, including their ability to penetrate, proliferate within, and ultimately kill *Drosophila* through various mechanisms. The effectiveness of EPF in pest management stems from their specific targeting of *Drosophila* while minimizing adverse effects on non-target organisms and the environment. EPF offer a promising alternative to chemical pesticides, contributing to sustainable pest control strategies. Continued research into optimizing EPF formulations and application methods will enhance their efficacy and integration into pest management programs. By leveraging the unique biological interactions of EPF, we can advance toward more environmentally friendly and effective solutions for controlling *Drosophila* populations and reducing the reliance on chemical insecticides.
